# Sorting of spatially incoherent optical vortex modes

**DOI:** 10.1038/s41598-020-59428-y

**Published:** 2020-02-13

**Authors:** Daniel O. Bezerra, Joao P. Amaral, Eduardo J. S. Fonseca, Cleberson R. Alves, Alcenísio J. Jesus-Silva

**Affiliations:** 1 Instituto de Física, Universidade Federal de Alagoas, P.O. Box 2051, Maceió, AL 57061−970 Brazil; 2 Grupo de Óptica, Faculdade Independente do Nordeste, Fainor, Vitória da Conquista, 45.055-030 Brazil

**Keywords:** Applied optics, Atmospheric optics, Fibre optics and optical communications

## Abstract

Coherent optical vortices have promising applications in quantum and classical optical communication. They add new degrees of freedom to code information. In this context, to implement a tool enabling sorting of spatially multiplexed vortex states is fundamental. By other hand, spatially incoherent vortices can be more robust in propagation through noise media, such as turbulent atmosphere or obstacles that block part of the light. Therefore, in this work we propose directly applying a high-resolution sorting scheme to spatially incoherent vortex states.

## Introduction

Since the discovery of the orbital angular momentum (OAM) of light or optical vortices^1^, one application that have attracted more attention recently is its use as a new degree of freedom to burst optical communications in the classical^2,3^ and quantum regimes^4,5^. Several orthogonal modes can be exploited to spatial multiplexing and increase the transmission capacity in optical communication systems. However, the most used optical beam is the OAM beam called Laguerre-Gauss (LG) beam^6^ which can be decomposed in terms of orthogonal components allowing being space-divided with low inter-modal crosstalk among multiple modes^2,3,7^. This beam has a finite spatial extend and due to its azimuthal phase dependence can be sorted^8–10^, being ideal to optical communications.

There are several proposals for OAM sorting. For example, the interferometry methods^11,12^, which require increasing complexity of the scheme with increasing number of modes to be sorted. However, the best approach to OAM sorting is based on a ray-optics coordinate transformation. To date, there are two types of such mode sorting. The first is based on log-polar transformation^13^ which can map OAM modes with an azimuthal phase to plane waves with tilts proportional to the vortex topological charge (TC)^8^. This approach is simple and efficient, but there is a limited separation of adjacent modes. One work improved this result at the cost of adding an additional phase-only hologram known as fan-out element that makes copies of the input optical fields and the interference of these copies can produce a better resolution^9^, but it also increase the complexity of the scheme. The second type of ray-optics coordinate transformation is the spiral transformation that maps spirals to parallel lines. In this case, the tilt of the plane waves is theoretically unlimited and, in practice, the resolution of the mode separation is considerably improved^10^.

Spatially incoherent optical vortices, are vortices originated by passing an incoherent light through a hologram designed to generate optical vortices^14^ or when a coherent optical vortex is scattered by a rough surface^15–18^. Those works have shown that the generated light presents a vortex in the coherence function, the so-called coherence vortex^19^. Remarkable, the lower coherence vortices are less influenced than those of higher coherence during propagation through turbulent atmosphere^20^ and through obstacles^21^. Therefore, discriminating incoherent optical vortices with different superimposed TCs is a topic of current interest for optical communication through turbulent atmosphere^22,23^.

## Results

### Concept and principle

**Figure 1:**
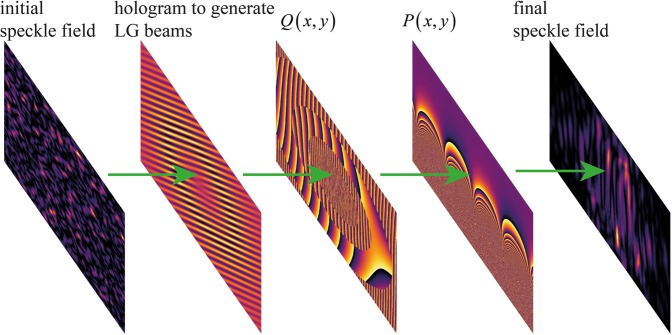
The general idea of the proposal: a spatially incoherent light represented by a speckle field, instead of coherent light, is used to generate OAM modes. The incoherent modes are passed through a usual system of coherent mode sorting and a final speckle field is produced from which we have to extract the information of the sorted modes.

The spiral transformation method has been successfully applied for high resolution sorting of coherent OAM modes of light^10^. In this work we have applied it to OAM mode sorting of spatially incoherent OAM modes. Figure 1 illustrates the main idea of the present work. A spatially incoherent light, which actually is a speckle field, is used with a hologram designed to generate LG beams. Therefore, incoherent LG beams are generated and their optical Fourier transform^24^ is projected over the transformation phase mask $Q(x,y)$. The resulting field has its optical Fourier transform projected over the correction phase mask $P(x,y)$. A final optical Fourier transform generates a final speckle field. The intensity of the final speckle field fluctuates randomly, but it still has the information of the sorted modes. This information is recovered from the correlations of the spatial intensity fluctuations^25^.

The spiral transformation is generated by a phase mask, which is described by the following equation^10^,

1 $$Q(x,y)=\frac{k\beta }{f({a}^{2}+1)}[(ax+y)\mathrm{ln}(\frac{r}{{r}_{0}})+(x-ay)\theta -(ax+y)],$$

where $k=2\pi /\lambda$ and $\lambda$ is the light wavelength, $f$ is the focal length of a lens put at a distance $f$ behind the phase element to perform its Fourier transform^24^, and ${a}$, $\beta$ and ${r}_{0}$ are constants. The $r$ and $\theta$ are the polar coordinates but the variable $\theta$ is not the conventional one since it is unlimited,

2 $$r={({x}^{2}+{y}^{2})}^{1/2},\theta ={\theta }_{0}+2m\pi ,$$

where

3 $${\theta }_{0}={\tan }^{-1}(\frac{y}{x})\in [0,2\pi ),m=\lfloor \frac{1}{2\pi a}\,\mathrm{ln}(\frac{r}{{r}_{0}}{e}^{-a{\theta }_{0}})\rfloor $$

and $\lfloor \rfloor$ is the floor function. A second phase element is used as a phase-correction mask such that its local gradient at a point (*u; v*) compensate the slope of the ray arriving at this point from the corresponding point (*x; y*) in the previous phase element^10^. This phase-correction element is given by,

4 $$P(u,v)=\frac{k{r}_{0}}{f}\frac{\beta }{1+{a}^{2}}\exp (\frac{au+v}{\beta })[\sin (\frac{u-av}{\beta })+a\,\cos (\frac{u-av}{\beta })]$$

where the constants are the same defined for the $Q(x,y)$ function and $(u,v)$ are the spatial coordinates at the plane of the phase-correction element.

### Experimental setup

**Figure 2:**
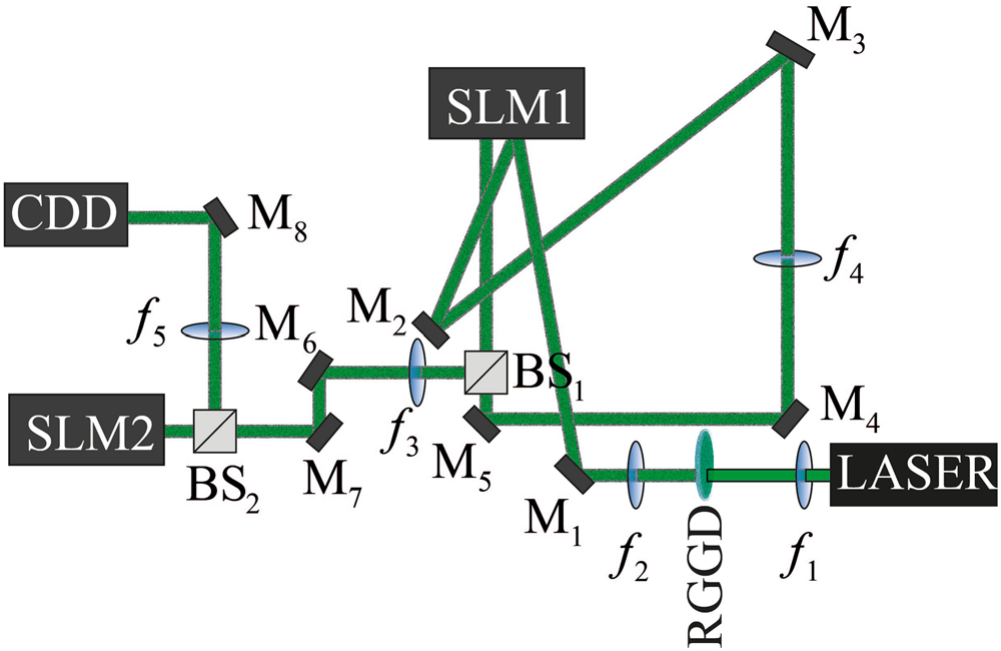
Experimental setup: $SL{M}_{1}$ and $SL{M}_{2}$ are spatial light modulators, *CCD* stands for charge coupled device camera, ${M}_{1}$ to ${M}_{8}$ are mirrors, $B{S}_{1}$ and $B{S}_{2}$ are beam splitters, the focal length of the lenses are ${f}_{1}=2.8\,mm$, ${f}_{2}=150\,mm$, ${f}_{3}=300\,mm$, ${f}_{4}=1000\,mm$, ${f}_{5}=200\,mm$.

The Fig. 2 displays the used experimental setup. A laser beam is expanded and collimated by a telescope formed through lenses specified by its focal length ${f}_{1}=2.8\,mm$ and ${f}_{2}=150\,mm$, where a rotating ground glass disc (RGGD) is placed between these lenses near the focus of the lens of focal length ${f}_{1}$. In order to get the best mode distinguishability we make use of the control of the source size^26,27^ slightly dislocating the RGGD from the focus. The obtained light illuminates the right half of $SL{M}_{1}$ which was half divided in order to contain, in the right, the hologram to generate LG beams^28^ and, in the left, the $Q(x,y)$ phase. The generated LG beam is Fourier transformed and projected over $Q(x,y)$ by a lens of focal length ${f}_{4}=1000\,mm$.

After the second time reflecting in the $SL{M}_{1}$ the LG beams acquire a phase $Q(x,y)$ and a lens of focal length ${f}_{3}=300\,mm$ projects the Fourier transform of this new field over the $SL{M}_{2}$ which contain the correction phase $P(u,v)$. A lens of focal length ${f}_{5}=200\,mm$ projects the Fourier transform of the field reflected from the *SLM*_2_ on the CCD camera. To calculate the phase elements $Q(x,y)$ and $P(u,v)$ we have used $f={f}_{5}=200\,mm$, $a=\mathrm{ln}(1.6)/(2\pi ),$ $\beta =1.8/(2\pi )mm$ and ${r}_{0}=1.1\,mm$.

### Theory

We present the theoretical formulation of the conceptual idea of the experiment with spatially incoherent light. The speckle field ${E}_{1}({{\bf{r}}}_{1})$ generated in the RGGG hits the first half of the SLM1 containing a hologram that encode the signal field $S({{\bf{r}}}_{1})$, i.e., LG modes. The resulting field is Fourier transformed by a lens of focal length ${f}_{4}$,

5 $${E}_{2}({{\bf{r}}}_{2})=\int {E}_{1}({{\bf{r}}}_{1})S({{\bf{r}}}_{1})\exp (\frac{2\pi i}{\lambda {f}_{4}}{{\bf{r}}}_{1}\cdot {{\bf{r}}}_{2})d{{\bf{r}}}_{1}.$$

The field ${E}_{2}({{\bf{r}}}_{2})$ is projected in the second half of the SLM1 containing the phase $Q({{\bf{r}}}_{2})$. The resulting field is Fourier transformed by a lens of focal length ${f}_{3}$,

6 $${E}_{3}({{\bf{r}}}_{3})=\int {E}_{2}({{\bf{r}}}_{2})\exp [iQ({{\bf{r}}}_{2})]\exp (\frac{2\pi i}{\lambda {f}_{3}}{{\bf{r}}}_{2}\cdot {{\bf{r}}}_{3})d{{\bf{r}}}_{2}.$$

The field in Eq. (6) hits the SLM2 which contains the phase $P({{\bf{r}}}_{3})$. The resulting field is Fourier transformed by a lens of focal length ${f}_{5}$ producing the final field at the CCD plane,

7 $${E}_{4}({{\bf{r}}}_{4})=\int {E}_{3}({{\bf{r}}}_{3})\exp [iP({{\bf{r}}}_{3})]\exp (\frac{2\pi i}{\lambda {f}_{5}}{{\bf{r}}}_{3}\cdot {{\bf{r}}}_{4})d{{\bf{r}}}_{3}$$

The reference field $R({{\bf{r}}{\boldsymbol{{\prime} }}}_{1})$ is just a Gaussian beam codded in the hologram and it propagates through the same optical components. In order to allow an analytical calculation of the correlation function we suppose, without loss of generality, that the for this field the transformations phases are $Q({{\bf{r}}{\boldsymbol{{\prime} }}}_{2})=P({{\bf{r}}{\boldsymbol{{\prime} }}}_{3})=0$. Therefore, the correlation function is written as^25^,

8 $$\langle {E}_{4}^{\ast }({{\bf{r}}}_{4}){E{\prime} }_{4}({{\bf{r}}{\boldsymbol{{\prime} }}}_{4})\rangle =\int {S}^{\ast }({{\bf{r}}}_{1})R({{\bf{r}}{\boldsymbol{{\prime} }}}_{1})\langle {E}_{1}^{\ast }({{\bf{r}}}_{1}){E}_{1}({{\bf{r}}{\boldsymbol{{\prime} }}}_{1})\rangle \exp [-iQ({{\bf{r}}}_{2})]\exp (-\frac{2\pi i}{\lambda {f}_{3}}{{\bf{r}}}_{2}\cdot {{\bf{r}}}_{3})\times \exp [-iP({{\bf{r}}}_{3})]\exp (-\frac{2\pi i}{\lambda {f}_{5}}{{\bf{r}}}_{3}\cdot {{\bf{r}}}_{4})\exp (\frac{2\pi i}{\lambda {f}_{3}}{{\bf{r}}{\boldsymbol{{\prime} }}}_{2}\cdot {{\bf{r}}{\boldsymbol{{\prime} }}}_{3})\exp (\frac{2\pi i}{\lambda {f}_{5}}{{\bf{r}}{\boldsymbol{{\prime} }}}_{3}\cdot {{\bf{r}}{\boldsymbol{{\prime} }}}_{4})\times \exp (-\frac{2\pi i}{\lambda {f}_{4}}{{\bf{r}}}_{1}\cdot {{\bf{r}}}_{2})\exp (\frac{2\pi i}{\lambda {f}_{4}}{{\bf{r}}{\boldsymbol{{\prime} }}}_{1}\cdot {{\bf{r}}{\boldsymbol{{\prime} }}}_{2})d{{\bf{r}}}_{1}d{{\bf{r}}}_{2}d{{\bf{r}}}_{3}d{{\bf{r}}{\boldsymbol{{\prime} }}}_{1}d{{\bf{r}}{\boldsymbol{{\prime} }}}_{2}d{{\bf{r}}{\boldsymbol{{\prime} }}}_{3},$$

where the symbol “*” stands for the complex conjugated field and $\langle \mathrm{..}.\rangle$ means an ensemble average.

We consider that the speckle fields are delta-correlated, i.e., its cross-correlation at the plane of the hologram that generates the signal and reference fields is $\langle {E}_{1}^{\ast }({{\bf{r}}}_{1}){E}_{1}({{\bf{r}}{\boldsymbol{{\prime} }}}_{1})\rangle =\delta ({{\bf{r}}}_{1}-{{\bf{r}}{\boldsymbol{{\prime} }}}_{1})$. Therefore, the integral in ${{\bf{r}}}_{1}$ and ${{\bf{r}}{\boldsymbol{{\prime} }}}_{1}$ can be evaluated resulting in

9 $${\tilde{S}}^{\ast }({{\bf{r}}}_{2}-{{\bf{r}}{\boldsymbol{{\prime} }}}_{2})\ast \tilde{R}({{\bf{r}}}_{2}-{{\bf{r}}{\boldsymbol{{\prime} }}}_{2})=\int {S}^{\ast }({{\bf{r}}}_{1})R({{\bf{r}}}_{1})\exp (-\frac{2\pi i}{\lambda {f}_{4}}{{\bf{r}}}_{1}\cdot ({{\bf{r}}}_{2}-{{\bf{r}}{\boldsymbol{{\prime} }}}_{2}))d{{\bf{r}}}_{1}.$$

Equation (9) represents a convolution between the Fourier transformed sign ${\tilde{S}}^{\ast }({{\bf{r}}}_{2}-{{\bf{r}}{\boldsymbol{{\prime} }}}_{2})$ and reference fields $\tilde{R}({{\bf{r}}}_{2}-{{\bf{r}}{\boldsymbol{{\prime} }}}_{2})$. Since the waist size of the reference beam is bigger than that of the signal beam, we can make the approximation $\tilde{R}({{\bf{r}}}_{2}-{{\bf{r}}{\boldsymbol{{\prime} }}}_{2})\approx \delta ({{\bf{r}}}_{2}-{{\bf{r}}{\boldsymbol{{\prime} }}}_{2})$ implying that ${\tilde{S}}^{\ast }({{\bf{r}}}_{2}-{{\bf{r}}{\boldsymbol{{\prime} }}}_{2})\ast \tilde{R}({{\bf{r}}}_{2}-{{\bf{r}}{\boldsymbol{{\prime} }}}_{2})\approx {\tilde{S}}^{\ast }({{\bf{r}}}_{2}-{{\bf{r}}{\boldsymbol{{\prime} }}}_{2})$. We also note that the integral in ${{\bf{r}}{\boldsymbol{{\prime} }}}_{3}$ in Eq. (8) results $\delta ({f}_{5}{{\bf{r}}{\boldsymbol{{\prime} }}}_{2}+{f}_{3}{{\bf{r}}{\boldsymbol{{\prime} }}}_{4})$. Using all of these results Eq. (8) simplifies to,

10 $$\langle {E}_{4}^{\ast }({{\bf{r}}}_{4}){E{\prime} }_{4}({{\bf{r}}{\boldsymbol{{\prime} }}}_{4})\rangle =\int {\tilde{S}}^{\ast }({{\bf{r}}}_{2}+{{\bf{r}}{\boldsymbol{{\prime} }}}_{4}\frac{{f}_{3}}{{f}_{5}})\exp [-iQ({{\bf{r}}}_{2})-iP({{\bf{r}}}_{3})]\times \exp (-\frac{2\pi i}{\lambda {f}_{4}}{{\bf{r}}}_{2}\cdot {{\bf{r}}}_{3})\exp (-\frac{2\pi i}{\lambda {f}_{4}}{{\bf{r}}}_{3}\cdot {{\bf{r}}}_{4})d{{\bf{r}}}_{2}d{{\bf{r}}}_{3}.$$

Using ${{\bf{r}}}_{4}={\bf{r}}$ and ${{\bf{r}}{\boldsymbol{{\prime} }}}_{4}=0$ we realize that the correlation function $\langle {E}_{4}^{\ast }({\bf{r}}){E{\prime} }_{4}(0)\rangle$ is identical with the result that could be obtained for the coherent fields that arrives at the final plane at the CCD^10^. There is no analytical result for the integral in Eq. (10) therefore, in the next section, we present a numerical simulation which is equivalent to the effect described by this integral.

### Numerical simulation and experimental results

For the numerical simulation we have used the band-limited angular spectrum method^29^ to propagate in the free space between the lenses and the SLMs, where each lens of focal length ${f}_{i}$ is represented by a quadratic phase $\exp [-ik{r}^{2}/(2{f}_{i})]$. Therefore, the field is multiplied by this quadratic phase to simulate the crossing of a lens. Similarly, the beam is multiplied by a phase $\exp [iQ(x,y)]$ or $\exp [iP(x,y)]$ to simulate the reflection of the field in the SLMs. We have first to generate a speckle field which is multiplied by the LG beams and then propagated though the lens of focus ${f}_{4}$ until the transformation phase $Q(x,y)$, and after that it follows the path through the lens of focus ${f}_{3}$ until the correction phase $P(x,y)$, and then through the lens ${f}_{5}$ until the CCD camera. To generate a speckle field we have multiplied a Gaussian function by a random phase and performed a Fourier transform of it. The size of the speckles where very small such that we can consider the field as a delta-correlated field. The reference beam is generated just passing a zero order LG beam through the system.

In the experiment, we measured the signal intensity followed by the reference intensity with RGGD stopped and then rotate the RGGD a small angle and stop it again to measure the next pair of signal and reference beams. For each pair of measurement we need to calculate the cross-correlation between the measured signal and reference intensities and we average over 100 measurements. Thanks to the Reed’s momentum theorem^30^ the intensity cross-correlation $\Gamma$ can be written as^31,32^,

11 $$\Gamma =A+{|\langle {E}_{4}^{\ast }{E{\prime} }_{4}\rangle |}^{2},$$

where $A$ is a background and $W=\langle {E}_{4}^{\ast }{E{\prime} }_{4}\rangle$ is given by Eq. 10.

**Figure 3:**
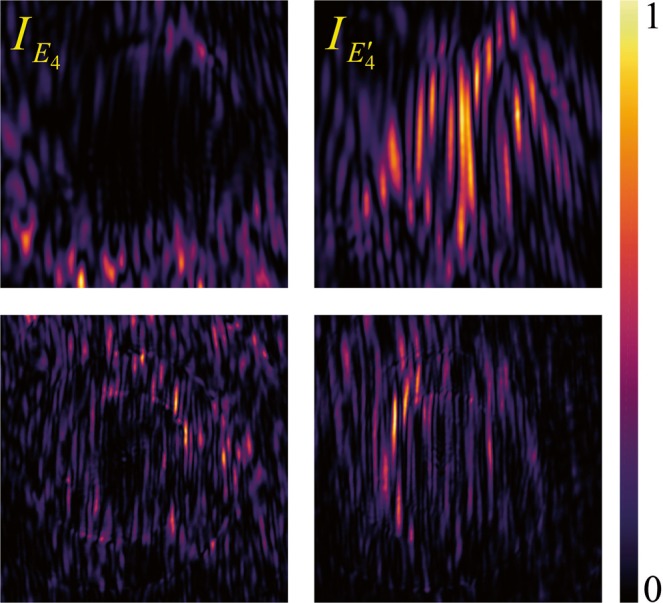
Random intensities patterns observed for the signal ${I}_{{E}_{4}}={|{E}_{4}|}^{2}$ ($m=2$, first column) and reference beams ${I}_{{E{\prime} }_{4}}={|{E{\prime} }_{4}|}^{2}$ ($m=0$, second column) at the detection plane. First line simulation and second line experiment. The windows are squares of $1.4\,mm\times 1.4\,mm$.

Figure 3 shows samples of the numerically calculated (first line) and experimentally measured speckle patters (second line), for the signal (first column) and reference beams (second column) at the CCD plane.

**Figure 4:**
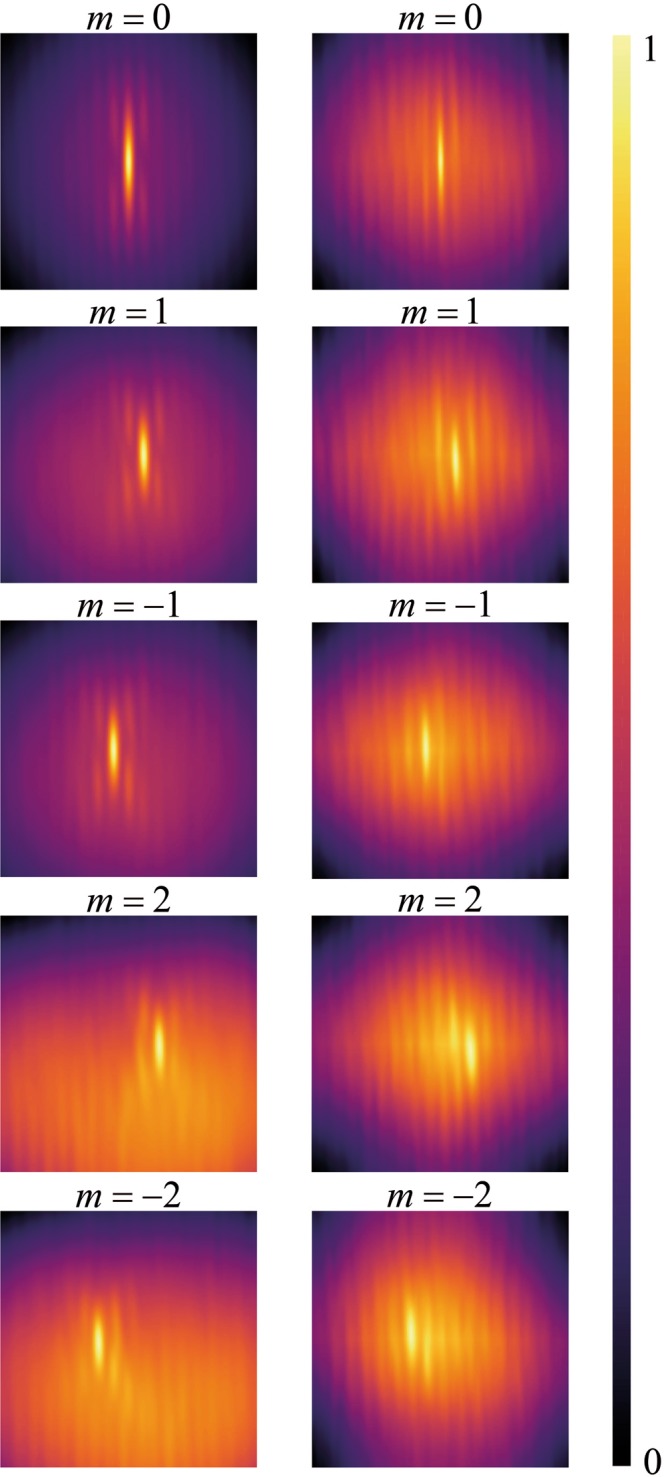
Simulation (first column) and experimental results (second column) for the intensity cross-correlation functions $\Gamma$. The windows are squares of $1\,mm\times 1\,mm$.

Figure 4, in the first and second columns, presents the results for the intensity cross-correlation patterns $\Gamma$ for the simulation and experiment, respectively. Although the signal and reference beams fluctuates randomly, the correlation function between them tends to a well-defined pattern in agreement with the prediction of the theory. We can observe that another prediction of the theory is also confirmed, i. e., despite of a background, the patterns for the cross-correlation ${|W|}^{2}$ was the same that the intensities of the coherent fields in ref. ^10^.

**Figure 5:**
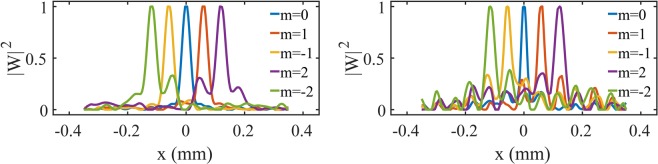
Profiles for the simulation (fist column) and experiment (second column) for the field cross-correlation function ${|W|}^{2}$.

Figure 5 shows transversal profiles taken at the center of the simulation (first column of Fig. 4) of the cross-correlation functions of the spatially incoherent field, and the same for the experimental results (second column of Fig. 4). In the experiment and simulation of the cross-correlation functions we have averaged over 100 calculations of the cross-correlation through 100 realization of the pair of random pattern (see Fig. 2) and have performed a background subtraction^33^, resulting ${|W|}^{2}$. The distance between two adjacent peaks is $\lambda {f}_{5}/(2\pi \beta )=59.1\mu m$^10^. The distance between adjacent peaks obtained directly by the image in the CCD camera is $59.5\mu m$, close to that value. Using this distance we can extract the topological charge from the distance of each mode to the central zero order mode. Therefore, the only previous calibration that we have to do is determining the position the zero order mode. We observe a good agreement between simulation and experiment indicating that the sorting scheme is viable with spatially incoherent light.

## Conclusion

We have observed that it is possible the use the spatial degrees of freedom of the coherence function to spatially multiplex coherence vortex to encode information for optical communications. Besides we note that it is possible to implement the sorting of spatially multiplexed spatially incoherent vortex states. Considering that spatially incoherent optical vortices beams are robust against scattering by the atmospheric turbulence and opaque obstacles, it may constitute a promising alternative for codding and transmitting information.

## Methods

### Devices in the experimental setup

A have used a laser model Ultralasers MSL-FN-532-200mW operating at 532 nm. The SLM_1_ used in the experiments is a Holoeye LETO and the SLM_2_ is a Hamamatsu X10468-01, both are phase-only SLMs based on reflective LCOS microdisplays. The Holoeye LETO SLM have a spatial resolution of 1920 × 1080 pixels and a pixel size of $6.4\,\mu m$ and the Hamamatsu SLM have a spatial resolution of 600 × 792 pixels and a pixel size of $20\,\mu m$. The resolution of the phase patterns displayed on the Holoeye SLM are 960 × 960 pixels, square shape and displayed side by side at the center. The resolution of the phase pattern displayed on the Hamamatsu SLM is 600 × 600 pixels displayed at the center. The camera used in the experiment is a PixeLink PL-B781F.
